# How Does Conditioned Pain Modulation Influence Motor Imagery Processes in Women with Fibromyalgia Syndrome? A Cross-Sectional Study Secondary Analysis

**DOI:** 10.3390/jcm13237339

**Published:** 2024-12-02

**Authors:** Víctor Riquelme-Aguado, Silvia Di-Bonaventura, María Elena González-Álvarez, Alazne Zabarte-Del Campo, Josué Fernández-Carnero, Antonio Gil-Crujera, Francisco Gómez-Esquer, Jorge Hugo Villafañe

**Affiliations:** 1 Department of Basic Health Sciences, Rey Juan Carlos University, 28933 Madrid, Spain; antonio.gil@urjc.es (A.G.-C.); francisco.gomez.esquer@urjc.es (F.G.-E.); 2 Grupo de Investigación Consolidado de Bases Anatómicas, Moleculares y del Desarrollo Humano de la Universidad Rey Juan Carlos (GAMDES), 28922 Alcorcón, Spain; 3 Fisioterapia Oreka CB, 45200 Illescas, Spain; alazne_1990@hotmail.es; 4 Cognitive Neuroscience, Pain and Rehabilitation Research Group (NECODOR), Faculty of Health Sciences, Rey Juan Carlos University, 28922 Alcorcón, Spain; silvia.dibonaventura@urjc.es (S.D.-B.); josue.fernandez@urjc.es (J.F.-C.); 5 Escuela Internacional de Doctorado, Rey Juan Carlos University, 28008 Madrid, Spain; 6 Department of Physical Therapy, Occupational Therapy, Rehabilitation and Physical Medicine, Rey Juan Carlos University, 28032 Madrid, Spain; 7 Musculoskeletal Pain and Motor Control Research Group, Faculty of Sport Sciences, Universidad Europea de Madrid, 28670 Villaviciosa de Odón, Spain; mail@villafane.it; 8 Department of Physiotherapy, Faculty of Sport Sciences, Universidad Europea de Madrid, 28670 Villaviciosa de Odón, Spain

**Keywords:** fibromyalgia, conditioned pain modulation, motor imagery, laterality discrimination, limb laterality judgement, chronic pain, pain neuroscience, descending pain modulation, mechanical hyperalgesia

## Abstract

**Background/Objectives**: Fibromyalgia syndrome (FMS) is a multifactorial pain syndrome not only characterized by widespread pain as the primary symptom but also accompanied by physical, psychological, and cognitive manifestations. Impairments in conditioned pain modulation (CPM) are common in this population; however, there is significant heterogeneity in the CPM response among women with FMS. The Left/Right Judgment Task (LRJT) is a validated method for studying motor imagery in chronic pain patients. Previous scientific evidence has not yet thoroughly investigated the relationship between CPM alterations and motor imagery processes in FMS patients. The aim of this study was to investigate the relationship between CPM and motor imagery. **Methods**: This is a secondary analysis of a cross-sectional study. Pain intensity (NPRS), disability (FIQ), mechanical hyperalgesia (PPT), descending pain modulation (CPM), and laterality discrimination (LRJT) were assessed in 30 women diagnosed with FMS. Participants were divided into two groups, responder and non-responder, according to their response to the CPM test. **Results**: Findings showed that the FMS subgroup of non-responders to CPM, performed worse in motor imagery processes (LRJT). Additionally, older age and higher mechanical hyperalgesia were also associated with poorer functioning of the inhibitory system. **Conclusions**: Women with FMS who are non-responders to CPM exhibit a reduced ability to perform motor imagery processes. Additionally, the non-responder group shown significant differences, such as older age and greater initial mechanical hyperalgesia compared to the responder group.

## 1. Introduction

Fibromyalgia syndrome (FMS) is a syndrome of widespread chronic pain characterized by a spectrum of psychological and physical symptoms [1,2], including fatigue [3], sleep disturbances [4], and cognitive dysfunctions [5]. It predominantly affects female populations and significantly impacts their quality of life. In Madrid, Spain, it is estimated that FMS affects up to 5% of women aged 46 to 60 years [6]. Diagnostic difficulties based solely on symptoms, the lack of identification of pathophysiological mechanisms responsible for the disease, and the complex therapeutic approach for FMS patients result in significant healthcare costs [7]. There is a growing body of research aimed at finding immunological biomarkers that may be linked to a state of neuroinflammation [8]. In deeper research into the potential aetiology of FMS, alterations in the neurobiological mechanisms of pain processing has been identified. It includes modifications in the descending pain modulation [9].

A relevant paradigm for evaluating these mechanisms is Conditioned Pain Modulation (CPM). This phenomenon implies that a primary painful stimulus can attenuate the perception of pain induced by a second stimulus, serving as an indicator of the functionality of the descending inhibitory system [10]. The efficacy of this process can indicate the endogenous ability of the nervous system to regulate pain [11,12]. However, in individuals with FMS, this mechanism is often compromised [13], which could explain the persistence and exacerbation of chronic pain in these patients. However, previous scientific literature has reported highly diverse results regarding CPM in patients with FMS [13,14,15]. There is significant variation in the protocols used to establish the CPM paradigm. This variability includes differences in the type of conditioning stimulus applied—such as thermal, mechanical, or pressure-based stimuli—as well as the specific anatomical region targeted for the stimulus or the application time. Moreover, responses to the CPM paradigm among FMS patients are inconsistent; while some FMS patients demonstrate a response to the conditioning stimulus, others show little to no response, indicating a range of individual differences in pain modulation among this population [16].

Concurrently, the Left/Right judgement task (LRJT) has been established as a valuable method in the study of motor imagery, especially in chronic pain patients [17]. This task involves recognizing and determining the orientation of images depicting various body parts, requiring individuals to mentally simulate or imagine the movement. This process is directly connected to motor imagery, as it engages the brain areas responsible for movement planning and execution. Additionally, the task relates to cortical representation, as it activates specific neural pathways associated with perceiving and understanding body orientation and positioning [18]. Poor performance in the LRJT in FMS patients suggests dysfunctions in the cortical representation of the body schema [19,20,21], which could be linked to the maladaptive pain mechanisms observed in these patients. Conversely, sensory information reaching the somatosensory cortex via the thalamus may become redundant, in part due to input from the posterior cord of the medial lemniscus associated with movement planning. It is proposed that this information could be influenced by pain, resulting in differences in movement planning between patients with chronic pain and those without pain [22]. The motor pathway may be modified by this incoming information, potentially leading to alterations in pain processing. Additionally, it is well established that the reorganization of the S1 cortex can contribute to dysfunctions in both motor production and motor learning [23,24].

Exploring the relationship between CPM and LRJT in the context of FMS holds significant scientific interest due to the potential link between dysfunctional pain modulation and deficits in somatosensory perception. The complex neurophysiological alterations observed in FMS patients may disrupt body schema representation and adversely affect motor imagery processes. Clarifying this relationship is valuable for both clinical and research purposes, as it could lead to more tailored therapeutic approaches. However, there remains a notable gap in the literature regarding FMS patient subgroups—specifically, CPM responders versus non-responders—and how these differences may impact motor imagery capabilities. Addressing this gap could greatly enhance our understanding of the pathophysiological alterations in FMS [25,26,27].

The objective of this study was to determine the relationship between CPM responsiveness and motor imagery performance, specifically in women with FMS. The secondary objective was to establish an association between CPM responses and the clinical and sociodemographic status of women with FMS. We hypothesize that those with impaired functioning of the descending inhibitory pain system will perform worse on laterality discrimination tasks, potentially revealing distinct patterns within the FMS population.

## 2. Materials and Methods

### 2.1. Study Design

This is a secondary analysis of assessments from a cross-sectional study [20] designed following STROBE guidelines [28]. Data were collected from February 2021 to December 2021. The study protocol received approval from the Ethical Review Board of Rey Juan Carlos University (2605202012920), in compliance with the Declaration of Helsinki, and all participants provided written informed consent before participating. All the tests were conducted by a researcher with over 10 years of clinical experience who was familiar with the participants’ study group.

### 2.2. Participants

The study sample consisted of 30 women with a formal diagnosis of FMS, recruited from the fibromyalgia association ‘AFINSYFACRO’ (Móstoles, Spain) through local support groups via advertisements and informational presentations. Inclusion criteria for FMS patients in this study were (1) a confirmed medical diagnosis of fibromyalgia, and (2) pain persisting for more than 3 months. Patients were excluded if they (1) had cognitive impairments that hindered their ability to comprehend or accurately complete any of the assessment variables, or (2) had prior experience with treatments or research involving laterality recognition of different body parts. A researcher ensured that participants comprehended the tasks and addressed any questions regarding the self-administered questionnaires. For the secondary analysis, women with FMS were divided into two subgroups based on their ability to respond to CPM. Group 1 (*n* = 14) included those women who exhibited negative or zero values in CPM. Group 2 (*n* = 16) comprised patients with FMS who demonstrated positive values in CPM.

### 2.3. Pain and Clinical Status

Pain intensity was assessed using the Numeric Pain Rating Scale (NPRS), a validated and widely used tool for self-reported pain evaluation in individuals with chronic pain. The NPRS operates on an 11-point scale, where 0 represents no pain and 10 represents the worst possible pain. NPRS scores are interpreted as follows: 0 = no pain, 1–3 = mild pain, 4–6 = moderate pain, and 7–10 = severe pain. Recent studies have strongly supported the use of the NPRS due to its high sensitivity and reliability in measuring pain levels, making it a preferred instrument in both clinical and research settings [29].

The impact of FMS on daily functioning and disability was evaluated using the Spanish version of the Fibromyalgia Impact Questionnaire (FIQ). This questionnaire consists of 10 items that evaluate the impact of FMS symptoms on daily functioning over the past week. The first four items assess physical function, overall well-being, and work performance, using Likert-type scales for responses. The remaining items employ a 10 cm visual analogue scale (VAS) to measure pain intensity, fatigue, sleep quality, stiffness, and psychological symptoms such as anxiety and depression. Likert scores are converted to a 0–10 range, while VAS scores are recorded directly. The total score is obtained by summing the individual item scores, with a possible range of 0 to 100. Scores from 0 to 39 indicate mild impact, 40 to 59 reflect moderate impact, and 60 to 100 signify severe impact. This questionnaire has been validated in the Spanish population, demonstrating high sensitivity, specificity, and internal consistency, making it a reliable instrument for assessing the impact of FMS [30].

Mechanical hyperalgesia was evaluated using the pressure pain threshold (PPT), defined as the minimum pressure necessary to provoke a pain sensation under standardized conditions. This measurement serves as a reliable indicator of pain sensitivity. A digital algometer (Model FXD 10, Wagner Instruments, Greenwich, CT, USA) was employed to measure pressure in kg/cm^2^ on the right upper trapezius muscle. The pressure was gradually increased at a rate of one kilogram per second until the participant reported pain. Each participant underwent two measurements, with a 30-s interval between them, and the mean value was recorded as the final result. This methodology has demonstrated high intra-examiner reliability, with an intraclass correlation coefficient (ICC) of 0.97, and substantial inter-examiner reliability, with an ICC of 0.79, for the upper trapezius in a healthy population. In patients with FMS, this method has been validated for assessing mechanical hyperalgesia, yielding an ICC of 0.88. A PPT result below 4 kg/cm^2^ is indicative of mechanical hyperalgesia [31,32,33].

Additionally, the PPT was chosen as the test stimulus because pressure stimuli are regarded as more reliable compared to other modalities, such as heat pain thresholds. Furthermore, the CPM effect is most effectively interpreted using pain thresholds and stimuli of predetermined intensity as test stimuli. This approach allows for a more consistent evaluation of pain responses and enhances the reliability of the findings [34,35].

### 2.4. Conditioned Pain Modulation (Cold Pressor Test)

The cold pressor test protocol involved several distinct steps. Initially, a pressure pain threshold (PPT) measurement was taken from the upper right trapezius muscle, following the previously described methodology. Subsequently, the participant’s left arm was immersed in a water bath maintained at a constant temperature of 12 °C for a duration of 60 s or until they reported a pain intensity of 7/10 on the Numeric Pain Rating Scale (NPRS). After the cold exposure, a second PPT measurement was taken from the same location on the right trapezius muscle. The conditioned pain modulation (CPM) value was calculated by subtracting the PPT value obtained during the cold water immersion from the baseline PPT value measured before the exposure. In healthy individuals, it is expected that the second PPT measurement will indicate an increased pressure tolerance compared to the first measurement, suggesting that the conditioning stimulus effectively enhances pain tolerance. However, in populations with chronic pain, this pain modulation system may not function as effectively, leading to a reduced CPM response. The cold pressor test has shown good intra-session reliability within chronic pain populations, with an intraclass correlation coefficient (ICC) of 0.77, further supporting its validity as a reliable and effective tool for assessing pain modulation [36].

### 2.5. Left/Right Judgment Test (LRJT)

A series of 20 and 50 images representing left and right hands, as well as left and right feet, were displayed on the screen of an electronic device using specialized software (Neuro-orthopaedic Institute, Adelaide, Australia). The images included different rotations and views, either palmar or dorsal. The order of images was randomized. Each image was shown for a maximum of 10 s or until the participant responded. Patients were instructed to quickly and accurately indicate the side of the hand or foot by pressing the corresponding key on the screen, labelling it “Left” for the left side and “Right” for the right side. They had a time limit of 10 s per image to respond. An example image was provided to familiarize participants with the test, and they were advised not to move their limbs during the assessment. The reaction time (RT) in seconds and the percentage of accuracy (ACC) were recorded as outcome measures. The reliability of the Recognise online application was previously established in both pain and non-pain populations [37]. The intraclass ICC for reaction time was ICC = 0.63–0.75 for feet and ICC = 0.51–0.91 for the trunk. For accuracy in laterality recognition, the ICC was 0.61–0.77 for feet and 0.69–0.71 for the trunk [38]. Internal and external validity were confirmed prior to the application’s online launch. Trials used a panel of images with the labels “L” for left and “R” for right. The application underwent three tests, achieving 100% internal validity [39]. In this secondary analysis, we further explored the variable by differentiating results based on accuracy and reaction time for both the right and left sides of the body.

### 2.6. Data Analysis

The sample size for this study was determined using G*Power (version 3.1.9.7). A medium effect size (Cohen’s d = 0.5) was assumed, with a significance level of 0.05 and a desired statistical power of 0.80. The analysis used an independent samples *t*-test with a total of 30 participants divided into two groups (16 and 14 participants, respectively). Although a larger sample size could improve power, the chosen sample was justified by the exploratory nature of the study and practical constraints. Data were analysed using the Statistical Package for the Social Sciences (SPSS, version 28, IBM, Chicago, IL, USA). A 95% confidence interval (CI) was set, and statistical significance was determined based on *p*-values less than 0.05. The normality of the data was evaluated using the Shapiro–Wilk and Kolmogorov–Smirnov tests, which indicated that the sample did not follow a normal distribution. As a result, comparisons of age, NPRS, FIQ, pre- and post-stimulus PPT, CPM, and LRJT variables between groups were performed using the non-parametric Mann–Whitney U test for independent samples. A significance threshold of *p* < 0.05 was applied to all statistical tests in the analysis.

## 3. Results

### 3.1. Demographic Variables and Clinical Status

Group 1, comprising non-responders to CPM, included fourteen women with FMS with a mean age of 57.57 ± 11.34 years. In contrast, Group 2, consisting of responders to CPM, included sixteen women with FMS, with a mean age of 47.94 ± 10.61 years. Based on the Fibromyalgia Impact Questionnaire (FIQ) scores, both groups showed a severe disease impact, with mean scores of 88.38 ± 3.89 in Group 1 and 87.74 ± 3.65 in Group 2. Additionally, both groups reported moderate pain intensity levels on the NPRS, with means of 6.86 ± 1.91 in Group 1 and 5.69 ± 1.95 in Group 2. Furthermore, mechanical hyperalgesia was observed in both groups, as measured by the PPT in the right upper trapezius muscle, with mean values of 1.87 ± 0.26 in Group 1 and 2.17 ± 0.38 in Group 2. The detailed results are presented in Table 1.

### 3.2. Pressure Pain Threshold After Conditioning Stimulus and Conditioned Pain Modulation

Following the conditioning stimulus, the subsequent measurement of the PPT in the right upper trapezius muscle revealed that both groups continued to present mechanical hyperalgesia. The mean PPT scores were 1.77 ± 0.21 for Group 1 and 2.40 ± 0.39 for Group 2. Statistical analysis showed no significant differences between the groups for this variable (*p* = 0.276). Group 1 demonstrated negative CPM results (−0.09 ± 0.16), while Group 2 showed positive CPM results (0.22 ± 0.09). Consequently, patients in Group 1 were classified as non-responders, and those in Group 2 as responders to the CPM paradigm. Statistically significant differences were found in post-conditioning stimulus PPT (*p* < 0.001) and CPM values between the groups (*p* < 0.001). The detailed results are presented in Table 2.

### 3.3. Impairment of Right/Left Judgment

FMS patients who were non-responders to CPM (Group 1) showed worse performance in the laterality judgment of hand and foot images. Statistically significant differences were found for the following variables: ACC for left feet in the 20-image round (*p* = 0.016); ACC for left hands in the 50-image round (*p* = 0.006); ACC for right hands in the 50-image round (*p* = 0.002); ACC for left feet in the 50-image round (*p* = 0.04). The remaining results can be seen in Table 3

## 4. Discussion

Findings showed that the FMS subgroup of non-responders to CPM performed worse in motor imagery processes (LRJT). Additionally, older age and higher mechanical hyperalgesia were also associated with poorer functioning of the inhibitory system.

Regarding the sample characteristics of the present study, FMS patients in the non-responder group (Group 1) were significantly older than those in the CPM responder group (Group 2). Additionally, women in Group 1 reported higher pain intensities. PPT measurements suggest mechanical hyperalgesia in both groups, as values are below 4 kg/cm^2^, with the CPM non-responder group showing worse results [40,41]. These results suggest that the ability to modulate pain in FMS patients may be influenced by older age and a worse clinical pain profile. Both groups show high levels of disability, consistent with previous scientific literature on the Spanish population [30].

In the present secondary analysis, findings suggest that the subgroup of FMS patients who do not respond to CPM performs worse on the LRJT than FMS patients who respond to the CPM paradigm. Compared to previous studies, the present work advances current knowledge by subclassifying patients into CPM responders and non-responders. Additionally, a more in-depth analysis was conducted, differentiating the accuracy and reaction times for the right and left sides of the body. The greatest impairment in most measurement variables was found in the LRJT tests displaying a higher number of images (50). Cognitive alterations in information processing have been described in FMS patients [5], leading us to hypothesize that this may explain the differences in results between a higher and lower number of images. In a previous study by Martinez et al. [19], only 30 images were used, with mean scores of 75.38 for ACC and 2.58 s for RT. Their study did not investigate whether differences existed with a higher versus lower number of images or whether there were differences in accuracy and reaction time between the left and right sides of the body. Additionally, only the hand region was evaluated, which has extensive cortical representation in Penfield’s homunculus [42,43]. However, our findings also revealed impairments when foot images were shown, despite feet having a smaller cortical representation.

Compared to other chronic pain populations, previous scientific evidence is contradictory [44,45]. Some studies report impaired laterality recognition in patients with carpal tunnel syndrome [46], knee osteoarthritis [47], phantom limb pain [48,49,50], and complex regional pain syndrome [51], while no alterations in motor imagery processes were found in chronic low back pain [38]. The pathophysiological mechanisms of these other conditions are also characterized by impaired pain modulation [36,52,53]. A further limitation in previous scientific literature is the variability in LRJT protocols. There is significant heterogeneity in the tests, including differences in the number of images presented, the inclusion of a practice round, and whether a body region corresponding to the patient’s pain is displayed. The absence of a “gold standard” for assessing implicit motor imagery processes in chronic pain patients complicates the interpretation of results and limits their generalizability to a broader population. Understanding the relationship between pain modulation capacity and motor imagery processes could be valuable for gaining insight into potential alterations and exploring future therapeutic approaches for these populations.

Recent scientific evidence [54] suggests that CPM tests could serve as surrogate biomarkers in the management of FMS syndrome. Restoring impaired endogenous pain modulation mechanisms through targeted non-pharmacological interventions may support long-term clinical recovery in FMS patients. Implicit motor imagery has been used in other chronic pain populations as a neuroscience-based therapeutic tool for pain management [55]. In FMS patients, studies are needed to determine the potential analgesic effects of implicit motor imagery and its role in reversing changes in pain modulation. Findings from the present work suggest that such interventions should target non-responders to CPM, as they show greater impairment in LRJT compared to CPM-responsive FMS patients.

### 4.1. Applications for Clinical Practice

Understanding how CPM affects the ability to perform motor imagery can guide therapists in designing rehabilitation programs. Interventions that integrate motor imagery exercises could be utilized in the early stages of treatment or as adjunct therapy, particularly for non-responder patients to CPM. Its applicability in pain management is noteworthy, as implicit motor imagery processes do not evoke painful sensations in women with FMS [20,21]. We also know that using the Left-Right Judgement Task (LRJT) in FMS patients is safe, regardless of negative psychological symptoms such as emotional (anxiety and depression) and cognitive aspects (catastrophizing and fear of movement). According to previous literature, psychological variables do not affect LRJT outcomes in women with FMS [21].

Furthermore, the finding that older women with FMS exhibit a greater deterioration in the expected analgesic response allows for a better understanding of their prognosis and future evolution, considering the results of the present study. Additionally, women in the non-responder group exhibited baseline differences in pressure pain threshold (PPT) measurements before the conditioning stimulus, with values below 2 kg/cm^2^. This finding could predict poorer functioning of the inhibitory system.

### 4.2. Limitations

This study has several limitations outlined as follows. First, the investigator conducting the measurements was aware of the study group to which each participant belonged. Second, the sample size is small, and caution should be exercised when extrapolating the results to the general population. Additionally, this is a secondary analysis, and longitudinal data cannot be obtained to assess how the capacity for CPM, LRJT, and clinical status may vary in this population. Finally, participants continued their usual medication, which may have influenced the obtained results.

### 4.3. Future Research Lines

Our research group will continue investigating women with FMS along the following lines. Longitudinal studies will be designed to monitor variations in CPM and its relationship with clinical status in women with FMS. Randomized clinical trials will be conducted to determine the efficacy of treatments that include motor imagery in women with FMS. Observational studies will characterize the genetic profiles of patients with FMS and assess the relationship between genetic polymorphisms and alterations in CPM and motor imagery. It would also be valuable to evaluate age and gender-related differences in the LRJT outcomes of FMS patients. Finally, future studies are needed to determine the psychometric properties of the LRJT in FMS patients to support its use in research.

## 5. Conclusions

Women with fibromyalgia syndrome (FMS) who are non-responders to CPM exhibit greater impairment in their ability to perform motor imagery processes. Additionally, the non-responder group displayed statistically significant differences, including older age and greater initial mechanical hyperalgesia compared to the responder group. Motor imagery exercises could be used early in treatment or as adjunct therapy, especially for CPM non-responders.

## Figures and Tables

**Table 1:** Descriptive statistics in demographic measures and clinical status (*n* = 30).

Measures	Group 1 (*n* = 14)	Group 2 (*n* = 16)	*p* Value Independent Samples Mann–Whitney U-Test
Age	57.57 ± 11.34	47.94 ± 10.61	0.012 *
Pain (NPRS)	6.86 ± 1.91	5.69 ± 1.95	0.055
FIQ	88.38 ± 3.89	87.74 ± 3.65	0.324
PPT Pre-stimulus	1.87 ± 0.26	2.17 ± 0.38	0.010 *

Data are presented as mean (SD). Both FMS groups reported moderate clinical pain intensities on the Numeric Pain Rating Scale (NPRS), elevated disease impact levels measured by the Fibromyalgia Impact Questionnaire (FIQ), and mechanical hyperalgesia prior to receiving the conditioning stimulus, as assessed by the pressure pain threshold (PPT). * indicates *p* value < 0.05.

**Table 2:** Comparison between groups in pressure pain threshold after receiving conditioning stimulus measures and conditioned pain modulation results.

Measures	Group 1 (*n* = 14)	Group 2 (*n* = 16)	*p*-Value Independent Samples Mann–Whitney U-Test
PPT post	1.77 ± 0.21	2.40 ± 0.39	0.001 *
CPM	−0.09 ± 0.16	0.22 ± 0.09	0.001 *

This table shows differences between groups in the variables of post-conditioning stimulus PPT and the final CPM outcome. Both groups exhibited mechanical hyperalgesia and reduced CPM values; however, significant differences were observed between the groups, with Group 1 showing worse outcomes. * indicates *p* value < 0.05.

**Table 3:** Comparison between groups regarding the ability to discriminate the laterality of images of feet and hands.

Measures	Group 1 (*n* = 14)	Group 2 (*n* = 16)	*p*-Value Independent Samples Mann–Whitney U-Test
RT (s) hands left (20)	3.20 ± 1.23	3.03 ± 1.02	0.342
ACC (%) hands left (20)	85.71 ± 12.38	85.31 ± 10.56	0.462
RT (s) hands right (20)	2.27 ± 0.83	2.65 ± 1.18	0.164
ACC (%) hands right (20)	72.86 ± 15.77	80.63 ± 16.11	0.145
RT (s) feet left (20)	2.27 ± 0.56	2.74 ± 1.51	0.145
ACC (%) feet left (20)	80.71 ± 15.91	90.63 ± 6.80	0.016 *
RT (s) feet right (20)	2.52 ± 1.26	2.59 ± 1.26	0.436
ACC (%) feet right (20)	82.14 ± 21.81	87.5 ± 15.38	0.220
RT (s) hands left (50)	3.31 ± 0.77	3.10 ± 1.04	0.273
ACC (%) hands left (50)	79.29 ± 10.60	87.25 ± 5.31	0.006 *
RT (s) hands right (50)	2.80 ± 0.71	2.86 ± 0.92	0.421
ACC (%) hands right (50)	76.14 ± 10.50	82.88 ± 8.06	0.002 *
RT (s) feet left (50)	2.5 ± 0.56	2.75 ± 1.25	0.249
ACC (%) feet left (50)	80.86 ±9.56	87± 9.06	0.04 *
RT (s) feet right (50)	2.60 ± 0.60	2.66 ± 1.13	0.42
ACC (%) feet left (50)	82.43 ± 12.40	85.25 ± 9.65	0.24

This table presents the differences between non-responders and responders in the CPM FMS patient groups regarding performance in the laterality recognition task for foot and hand images. The variables studied were reaction time (RT) and accuracy (ACC) in rounds of 20 and 50 images. Statistically significant differences were considered for values of *p* < 0.05. * indicates *p* value < 0.05.

## Data Availability

Data are contained within the article.
